# Intensive, Multi‐Technology Rehabilitation Programme for Chronic Stroke Survivors: Outcomes of a Phase I Single‐Arm Repeated‐Measures Study

**DOI:** 10.1002/pri.70321

**Published:** 2026-08-19

**Authors:** Andrew Kerr, Madeleine Grealy, Milena Slachetka, Dwight Tse, Philip Rowe

**Affiliations:** ^1^ Biomedical Engineering University of Strathclyde Glasgow UK; ^2^ Psychology University of Strathclyde Glasgow UK

**Keywords:** chronic stroke, intensity, multi‐technology, rehabilitation

## Abstract

**Background:**

Effective rehabilitation interventions are needed to support recovery in people living with long‐term post‐stroke disability. Cocreation has been proposed as a method for enhancing the acceptability of technology‐supported rehabilitation and may improve rehabilitation outcomes.

**Objective:**

To observe changes in outcome following participation in a multi‐technology rehabilitation intervention for motor recovery in people with chronic stroke and to explore factors associated with differential response.

**Design:**

Pre–post, single‐arm intervention study.

**Methods:**

Participants with stroke ≥ 12 months previously and persistent motor impairment, completed an 8‐week, group‐based circuit class incorporating multiple rehabilitation technologies. Standardised measures of activity and participation were collected at baseline, pre‐intervention, and post‐intervention.

**Results:**

Sixty‐seven participants (mean age 60.49 ± 14.44 years; mean 41.87 ± 41.31 months post‐stroke) were enroled and 59 (88.06%) completed a mean of 18.52 ± 6.44 sessions. Eight did not complete, mainly due to transport or illness. Statistically significant (*p* < 0.05) changes from pre‐to post‐intervention were observed across all outcomes. Subgroup analysis showed that participants with poorer balance, upper‐limb function and global disability at baseline showed greater pre–post improvements than higher‐functioning participants.

**Conclusions:**

Improvements in mobility and global function were observed following participation in a cocreated, multi‐technology group rehabilitation programme for people with chronic stroke. Greater pre–post improvements were observed among participants with greater initial disability. These preliminary findings need confirmation in controlled studies.

**Trial Registration:**

Clinical Trial registration: NCT06787768.

## 1 Introduction

The UK's National Institute for Health and Care Excellence (NICE) recently changed its recommended guidance to include 3 h of multi‐disciplinary rehabilitation, every day, after a stroke, as a minimum (Excellence 2023). They also recommend that rehabilitation should continue for as long as it continues to achieve its goals. Delivering evidence based rehabilitation can improve recovery (Hubbard et al. 2012), reduce disability and improve general health (Urimubenshi et al. 2017). Delivering these guideline recommendations will not only benefit individuals, and their future health but will likely impact the estimated cost of social care (£19.3 billion), unpaid care (£24.4 billion) and lost productivity (£2.3 billion) caused by stroke in the UK (King et al. 2020). Given the National Health Service's (NHS's) historical inability to meet previous, more modest, recommendations for rehabilitation after stroke (Gittins et al. 2020) and generally poor, and slow, professional engagement with guidelines (Bernhardsson et al. 2014; Bird et al. 2019), the current NICE recommendations present a considerable challenge that may be unachievable within current budgets.

Integrating technology into rehabilitation practice creates many advantages for users, including improved capacity to self‐manage, and offers a realistic way of supporting individuals and services to meet guideline recommendations (Thompson et al. 2023). For example, mechanical assistance systems enable high repetition of movement practice, digital games can motivate and provide performance feedback on movement and extended reality technology can create the enriched environments considered important in stimulating recovery (Jones et al. 2010). However, there are organisational and technology design barriers to integrating technology more fully into practice, such as usability, institutional IT systems, set up costs (e.g., equipment and staff training), and evidence of efficacy and training (Sweeney et al. 2020).

The variable and complex nature of stroke presents a unique challenge for the development of rehabilitation technologies. Research and engineering have typically focussed on developing and evaluating devices to address single impairments, for example robotics for upper limb weakness, treadmills for gait training and digital apps for speech therapy. Stroke survivors, however, present with individual combinations of impairments that require individually tailored, multi‐technology, solutions.

Our multi‐disciplinary research group, in collaboration with the UK's NHS, charitable partners and a large (*n* = 100) group of people living with disability caused by stroke, have cocreated a multi‐technology enriched rehabilitation gym (TERG) that has been designed and tested for usability over thousands of rehabilitation hours (Kerr et al. 2023) to address the multiple impairments common after stroke (mobility, communication and cognitive). This resulted in a circuit‐based group delivery tailored to individual goals with minimal assistance for cost effectiveness.

A recently completed study (NCT05981729) demonstrated the feasibility of our model in an NHS stroke unit (Sweeney et al. 2025). Key findings showed that the model delivered an average of 52 minutes of additional rehabilitation every day without safety concerns and with supervision provided by rehabilitation assistants. The next step in developing this intervention is to investigate whether recovery‐related motor changes are observed following the intervention. Withholding rehabilitation is neither ethical nor reflective of usual practice, making a single group approach a pragmatic choice. While alternative designs such as wait‐list controls or crossover studies could be considered, these were beyond the scope of this initial work. Incorporating analyses such as clustering baseline characteristics offers a means to capture variation within the cohort, providing insights into how rehabilitation might be tailored to patient needs and help to inform future studies. This approach lays the groundwork for a more definitive evaluation of the model's impact.

The primary aim of this study was to gather preliminary evidence of response to the TERG intervention model in people living with motor disability caused by stroke, at least 12 months previously, using a single cohort, pre‐post study design. Secondly, to identify, through cluster analysis, baseline characteristics of the sample that could explain any observed variation in response.

## 2 Materials and Methods

Single‐arm study with two baseline assessments and post‐intervention follow‐up to assess the outcomes of an 8‐week, multi‐technology rehabilitation intervention on the function and independence of people in the chronic phase of stroke recovery. The study was registered with the National Library of Medicine (trial number NCT06787768) and was given ethical approval by the institutional ethics committee (UEC20/08).

Participants with a diagnosis of stroke, at least 12 months previously, that caused movement disabilities were invited to participate (see Table S1 for details). Participants were recruited through an open public process with the support of a medical charity (Chest Heart and Stroke Scotland). Recruitment strategies included announcements via social media platforms, coverage in national media outlets, and word‐of‐mouth. No restrictions were placed on who could respond, aside from the eligibility criteria.

### 2.1 Intervention

The intervention comprised an 8‐week rehabilitation programme delivered within a multi‐technology rehabilitation gym on a university campus (Kerr et al. 2023). The gym incorporated immersive and non‐immersive virtual reality, treadmills, an upper‐limb weight support system, a uniplanar upper‐limb robotic device, an interactive hand and forearm training system, interactive speech and cognitive training applications, and movement resistance and power‐assistance equipment (see Table S2 for details).

Participants attended group‐based circuit classes lasting 120 min. Each session was divided equally between two rehabilitation areas: one focussed on seated upper‐limb, speech and cognitive rehabilitation, and the other on balance, gait, strengthening and aerobic exercise.

The seated component began with 15 min of upper‐limb priming activities, including mirror therapy, sensory transcutaneous electrical nerve stimulation (TENS) and/or hand vibration. This was followed by approximately 40 minutes of technology‐assisted upper‐limb, speech and cognitive rehabilitation. Participants typically completed around four technology‐based activities during this period, although the exact combination and time spent at each activity varied according to individual preference and rehabilitation needs.

The second component began with 15 min of power‐assisted exercise, followed by approximately 40 min of technology‐assisted gait, balance, strengthening and aerobic exercise. As in the seated component, participants typically completed around four technology‐based activities, with progression through activities determined by participant engagement and therapist judgement rather than fixed time allocations.

The intervention was intentionally delivered as a bundled rehabilitation model in which multiple rehabilitation technologies and exercise approaches were integrated within a single programme rather than evaluated as isolated components. Individual rehabilitation programmes were designed, supervised and regularly reviewed by a physiotherapist according to participant‐identified goals, baseline impairments and ongoing performance. Equipment, task selection and progression were individualised throughout the programme. Task difficulty was progressed by adjusting movement amplitude, duration, movement speed, resistance, robotic assistance, balance challenge, virtual reality game difficulty and task complexity according to participant performance and tolerance. As participants became more familiar with the technologies and gained confidence, they were encouraged to take increasing ownership of their rehabilitation by selecting activities and determining the order and time spent at individual stations within the overall circuit structure.

Participants attended a minimum of two sessions per week, with the option of attending up to five sessions per week. This provided a minimum rehabilitation dose of 240 min per week (19.2 h over 8 weeks) with a maximum possible exposure of 600 min per week (80 h over 8 weeks).

### 2.2 Measures

A range of outcome measures across the International Classification of Functioning, Disability and Health (ICF) were included to capture the expected diverse impact of the intervention. These were selected for their measurement properties and acceptability in routine clinical practice.

Performance based measures were used for walking (10‐m Walk Test (10MWT) and Functional Ambulatory Category (FAC) (van Bloemendaal et al. 2012; Cheng et al. 2020)), sit to stand (5 times sit to stand, FTSTS (Mong et al. 2010)), arm (Action Research Arm Test (ARAT) (Hsieh et al. 1998) and grip strength (Bertrand et al. 2015)) and balance (Berg Balance Scale (BBS) (Blum and Korner‐Bitensky 2008)) functions. The modified Rivermead Mobility Index (mRMI) (Walsh et al. 2010) was added as a patient reported measure of physical capacity.

To capture information at the participation level, the Stroke Impact Scale (SIS16) was included, and this is a Patient‐Reported Outcome Measure which features both activity and participation questions (Hamid et al. 2025).

### 2.3 Data Collection Procedure

Outcome measures were recorded at three time points; (1) 2 weeks before the intervention commenced (baseline 1), (2) immediately before the intervention commenced (baseline 2) and (3) immediately after the intervention ended (outcome). The exceptions to this were the patient reported measures (mRMI and SIS16) and BBS, which were only recorded at time points 1 and 3 due to time constraints.

Baseline 2 was added to the data collection procedure to allow statistical comparison between the two baseline points. In the absence of a control group, this provided data on the stability of physical function measures.

### 2.4 Statistical Analysis

Missing data (10.9%) were assessed with Little's test and addressed using multiple imputation (20 datasets), with results pooled using Rubin's rules. Changes in outcomes were then investigated using multilevel modelling between baseline and outcome time points. Effect sizes for changes in outcome measures were reported using Cohen's *d*. Differences between the two baselines were tested for significance with a paired *t*‐test.

Intervention outcomes are rarely uniform across patients. To explore whether we could meaningfully divide the sample into clusters based on baseline performances, to account for these differences in improvement, two‐step cluster analyses were conducted on baseline measures. To simplify the cluster analyses, given that the performance indicators were likely correlated to each other, we conducted exploratory factor analysis (principal axis factoring with oblimin rotation) prior to the cluster analyses, to explore the domains of recovery, retaining factors based on eigenvalues > 1.0 and scree plot inspection.

## 3 Results

Recruitment ran for 20 months between April 2023 and December 2024. During this period, 89 individuals expressed an interest in participating. Sixty‐seven met the eligibility criteria and all provided informed consent. Five participants subsequently withdrew before commencing the intervention, leaving 62 participants who started the programme. Three participants withdrew before completing the intervention, leaving 59 participants who completed the programme and outcome measures and were included in the final analysis (Figure S1). Table 1 presents the characteristics of the consented sample.

**TABLE 1:** Characteristics of the 67 participants who consented to the study.

Variable	Average/count	Standard deviation (range)
Age	60.27	12.17 (25–82)
Gender	Male: 51/67 (76%)	
	Female: 16/67 (24%)	
Time since stroke (months)	41.87	41.31 (12–289)
MoCA	25.56	4.67 (6–30)
Aphasia	30/67	
Sessions attended (*n* = 59)	18.52	6.44 (10–38)
Total hours of rehabilitation, (*n* = 59)	37.05	12.89 (20–76)

The 59 participants who completed the programme attended an average of 2.28 (SD 0.81) sessions per week, equivalent to 18.52 (SD 6.44) sessions over the 8‐week intervention, corresponding to 37.05 (SD 12.89) hours of rehabilitation.

While improvements were observed across all outcomes (see Table 2), the greatest changes were observed for five times sit‐to‐stand (*d* = 0.61), grip (*d* = 0.49) and balance (BBS, *d* = 0.31), see Table 3. Only grip (MCID, 5–6 kg (Bohannon 2019), pooled difference 4.57 kg), 10MWT (MCID for speed 0.1–0.2 m/s (Bohannon and Glenney 2014), pooled speed difference 0.11 m/s) and FTSTST (MCID 0.7–3 s (Agustín et al. 2021), pooled difference 10.87 s) approximated to, or exceeded, reported MCIDs in this population. Exploratory analyses found no evidence of a linear association between programme attendance and changes in walking speed, grip strength, or FTSTS performance (all *r* values < 0.20).

**TABLE 2:** Means and standard deviations for measures at baseline (baseline 1, *n* = 67), 2 weeks later (baseline 2, *n* = 67) and after the programme (outcome, *n* = 59).

	Baseline 1	Baseline 2	Outcome
ARAT	25.65 (25.74)	26.01 (26.10)	26.90 (26.22)
GRIP	8.40 (9.32)	9.17 (10.01)	12.97 (10.38)
FAC	4.40 (0.92)	4.49 (0.96)	4.55 (0.69)
10MWT	23.80 (25.36)	24.13 (27.62)	18.93 (19.41)
5STS	26.07 (17.81)	22.89 (14.60)	15.21 (10.02)
mRMI	11.09 (3.29)		11.54 (3.10)
SIS16	59.96 (11.65)		63.11 (11.53)
BERG	43.69 (11.21)		47.11 (9.68)

**TABLE 3:** Statistical difference and effect size of change in outcome measures from baseline.

	Baseline versus outcome		
	Pooled difference	Pooled *p*‐value	Cohen's d
ARAT	1.25	0.010	0.048
GRIP	4.57	< 0.001	0.491
mRMI	0.45	0.028	0.136
SIS16	3.15	0.001	0.270
FAC	0.15	0.004	0.164
10MWT	4.87	< 0.001	0.192
5STS	10.87	< 0.001	0.610
BBS	3.43	< 0.001	0.306

There were minor, non‐significant, differences between the two baseline measures with the notable exception of FTSTS, which showed a clear difference (*p* = 0.006). The relatively small differences between baseline observations suggest a stable group with learning and familiarisation likely accounting for the small observed differences, see Table 2. The continued, and statistically significant, gains in grip, walking (10MWT) and STS (FTSTS), however, support the interpretation that changes were related to the intervention rather than natural recovery or test familiarity.

### 3.1 Cluster Analysis

For the exploratory factor analyses, all imputations yielded a two‐factor solution with no cross‐loading detected (Kaiser‐Meyer‐Olkin tests > 0.80 and *p* < 0.001 for Bartlett's tests of sphericity). We labelled the first factor, which included ARAT and Grip, as arm measures and the second factor, including mRMI, SIS16, FAC, 10MWT, FTSTS, and BBS, as balance and independence measures. Accordingly, for the cluster analysis, the first analysis used baseline arm measures as input and the second used baseline balance and independence measures as input. Both yielded two‐cluster solutions of acceptable quality (silhouette measures of cohesion > 0.70).

Arm clusters: Cluster 1 (*n* = 36) had lower baseline ARAT and grip scores than Cluster 2 (*n* = 31). No demographic differences were observed. Cluster 1 showed less grip strength improvement but greater gains in balance and independence.

Balance/independence clusters: Cluster 1 (*n* = 20) had poorer baseline balance and independence, and it was older and predominantly male than Cluster 2 (*n* = 47). Cluster 1 showed slightly less grip strength improvement but similar, or greater, gains in balance and independence, see Table 4 for details.

**TABLE 4:** Means and standard deviations for arm and balance clusters at baseline and outcome.

	Arm clusters			
	Cluster 1, *n* = 36		Cluster 2, *n* = 31	
	Baseline	Outcome	Baseline	Outcome
ARAT	2.53 (5.10)	3.29 (6.25)	52.51 (6.92)	54.32 (5.51)
GRIP	2.69 (3.56)	6.29 (4.48)	15.02 (9.70)	20.73 (9.95)
FAC	3.97 (1.06)	4.24 (0.78)	4.90 (0.30)	4.91 (0.31)
10MWT	34.22 (30.54)	26.97 (22.41)	11.69 (6.65)	9.59 (8.38)
5STS	33.83 (22.28)	18.51 (12.32)	17.06 (6.35)	11.37 (3.45)
mRMI	9.72 (3.21)	10.26 (3.19)	12.68 (2.62)	13.02 (2.27)
SIS_16	56.61 (11.43)	59.50 (11.29)	63.84 (10.78)	67.29 (10.64)
BBS	38.19 (11.56)	42.52 (10.54)	50.06 (6.33)	52.45 (4.41)

	**Balance clusters**			
	**Cluster 1, *n* = 20**		**Cluster 2, *n* = 47**	
	**Baseline**	**Outcome**	**Baseline**	**Outcome**
ARAT	5.1 (14.84)	5.61 (15.64)	34.40 (1.50)	35.96 (24.97)
GRIP	3.38 (6.53)	7.26 (8.25)	10.53 (9.58)	15.4 (10.32)
FAC	3.20 (0.77)	3.69 (0.58)	4.91 (0.28)	4.92 (0.29)
10MWT	49.38 (34.22)	39.64 (24.57)	12.91 (5.76)	10.12 (4.99)
5STS	44.75 (27.34)	20.95 (15.56)	18.12 (6.92)	12.76 (4.77)
mRMI	7.05 (2.70)	7.67 (2.38)	12.81 (1.50)	13.19 (1.42)
SIS_16	48.25 (9.03)	51.64 (8.75)	64.94 (8.56)	67.99 (8.84)
BBS	30.70 (9.65)	35.88 (9.33)	49.21 (6.12)	51.89 (4.38)

These exploratory analyses identified improvements across all measured motor outcomes over the intervention period. Taken together, poorer baseline function (of either factor) was associated with larger observed changes in balance and independence, whereas better baseline function was associated with larger changes in grip strength.

### 3.2 Adverse Events

No serious adverse events were recorded during the intervention period. Safety monitoring was undertaken throughout each session by the supervising physiotherapists, who monitored participants for symptoms, fatigue, pain, balance concerns and any difficulties completing activities. Minor adverse events were prospectively recorded and reviewed by the clinical team.

Seventeen minor adverse events occurred among participants during the intervention period. The most common events were post‐session fatigue (*n* = 6), joint pain (shoulder *n* = 2, knee *n* = 2), non‐injurious falls (*n* = 2), transient illness/cold symptoms (*n* = 2), and minor skin abrasions to the hand (*n* = 3). All events resolved within 7 days. No adverse events resulted in withdrawal from the programme. Where required, activity selection, intensity, or duration was modified to accommodate symptoms and support continued participation.

## 4 Discussion

The principal finding of this study was that participants with chronic stroke demonstrated generally modest, but statistically significant, pre–post improvements following participation in an 8‐week, multi‐technology rehabilitation programme. The magnitude of change varied across outcome measures, with the largest improvements observed in grip strength, walking speed and FTSTS, all of which approximated, or exceeded, published MCID values. In contrast, several other outcomes demonstrated only small effect sizes, emphasising that statistical significance does not necessarily correspond to clinically meaningful improvement.

These findings add to existing evidence suggesting that meaningful improvements may still be achievable in the chronic phase after stroke and demonstrate the feasibility of delivering a technology‐enriched rehabilitation programme with an 88.1% completion rate that is comparable to previous reports (Kerr et al. 2023; Sweeney et al. 2025). The observed improvements were consistent with findings from other circuit‐based interventions. In a circuit class therapy intervention with sub‐acute stroke patients, English et al. 2015 showed broadly similar improvement in walking speed (0.2 m/s) (English et al. 2015) and (Kim et al. 2016) demonstrated a statistically significant (*p* < 0.01) change in balance (Kim et al. 2016). Both these studies included a control group of dose matched usual care, which did not differ significantly from the experimental circuit‐based interventions, suggesting that the potential value of this multi‐technology approach may lie in its cost effectiveness and ability to translate into a self‐management model through existing and adapted community resources, rather than its superiority over usual care.

The greatest changes (*p* < 0.001 and MCID) were observed in three physical performance measures: grip strength, walking speed (10MWT) and sit to stand (FTSTS). One possible explanation for this is training specificity. Almost all the activities required grip (unimanual and bimanual) with some activities targeting grip specifically (e.g., GripAble), transferring between activities required moving from a seated to standing position and some activities specifically targeting sit to stand (e.g., functional squat). This interpretation is consistent with evidence supporting task‐specific training for recovery of upper and lower limb function (French et al. 2016). Achieving change in these single performance tests (e.g., FTSTS, grip and walking speed) may, of course, be easier than global measures of physical function such as the mRMI. While the Stroke Impact Scale‐16 demonstrated a statistically significant improvement (*p* < 0.001), the magnitude of change (3.15 points) was substantially below the reported minimal clinically important difference (9.4–14.1 points) (Pomeroy et al. 2016), suggesting limited clinical importance despite statistical significance and highlighting the difficulty affecting behavioural change, even when physical capacity improves.

The absence of an observed association between attendance and outcome change should be interpreted cautiously. Attendance represents only one component of rehabilitation dose and does not capture differences in exercise intensity, progression, or the specific activities undertaken by individuals. In addition, the sample size and heterogeneity in baseline impairments and response to training may have limited the ability to detect more subtle dose‐response relationships. These findings highlight the potential importance of a tailored approach to rehabilitation after stroke.

### 4.1 Exploring Heterogeneity in Pre‐Post Changes

Despite the moderate sample size, the large variation in effect sizes (Cohen's d) warranted subgroup analysis. This level of variation is a feature of rehabilitation research, particularly in stroke (Pomeroy et al. 2016) that requires a stratified approach to analysis; our approach was clustering. The exploratory cluster analysis identified two participant profiles characterised primarily by differences in baseline arm function. Participants with lower baseline ARAT and Grip scores (Cluster 1) demonstrated larger pre–post improvements in balance and global disability measures, raising the possibility that baseline functional profiles may influence the pattern of change observed following rehabilitation. In contrast, participants with higher baseline arm function demonstrated smaller changes in balance and disability measures but greater changes in grip‐related outcomes, potentially reflecting differences in baseline capacity or the distribution of room for improvement across measures. Although these cluster profiles are preliminary, and require replication in larger samples, they may help generate new hypotheses regarding the importance of tailoring rehabilitation goals to initial functional levels, that is patients starting with lower motor function may benefit more from holistic, mobility‐oriented interventions, while those with higher baseline function might benefit from more targeted upper‐limb therapy.

### 4.2 Clinical Implications

Across the 59 participants that completed the intervention, there was an average of 37 h, or 4.6 h per week, of rehabilitation; less than 1 h of additional rehabilitation per day. While this technology approach could undoubtedly contribute to achieving NICE guideline recommendations of 3 hours per day, it is clearly not the whole solution, requiring additional input from NHS provided rehabilitation and other strategies such as telerehabilitation.

A strength of this study is the diverse nature of the participants that resulted from the broad inclusion criteria and may better reflect the population seen in practice. Participants with greater baseline impairment demonstrated larger pre–post improvements than higher‐functioning participants. Although this observation may not surprise experienced clinicians, it provides preliminary support for further investigation of technology‐supported, and human‐supervised, rehabilitation for individuals with greater disability.

### 4.3 Limitations

In reporting these generally positive findings, the study limitations should be acknowledged. The similarity between the two baseline assessments suggests that participants were clinically stable before the intervention period. Without a control group, however, it remains impossible to determine whether the observed changes were attributable to the intervention rather than to other factors, and causal inferences, therefore, cannot be made. While natural recovery of physical function is less likely in a chronic stroke population, having a control group would have differentiated any improvement related to time from the intervention. The sample was not only moderate in size, biased towards younger and predominantly male, but the recruitment strategy is likely to have favoured more motivated individuals, all of which limits the generalisability of the findings. While the data clustering approach revealed some useful insights, the small size of the individual clusters should be considered when interpreting them and should only be used as hypothesis generating rather than testing.

As the intervention was delivered as a complex, multi‐component rehabilitation programme, it is not possible to determine which specific technologies, or exercise components, contributed to any observed changes. The findings therefore relate to the observed outcomes associated with the overall rehabilitation model rather than any individual technological modality.

A final design limitation was the lack of long term follow up data, having an immediate impact on function is important, but understanding if and for how long this is sustained would benefit rehabilitation planning.

### 4.4 Recommendations

Future studies should consider designs with true control groups, larger samples and longitudinal data capture to determine whether the observed gains are attributable to the intervention, and can be sustained, and whether cluster membership predicts long‐term outcomes as well as whether different intervention types or therapy intensities yield differential benefits across these identified clusters.

## 5 Conclusion

In this single‐arm study, improvements in mobility and global function were observed following participation in a cocreated, multi‐technology rehabilitation intervention designed to deliver recommended levels of rehabilitation after stroke. The largest effect sizes were observed for sit‐to‐stand performance, grip strength, and walking speed, with greater pre–post improvements among participants with greater initial disability. These findings should be confirmed in controlled studies.

## Author Contributions

Andrew Kerr, Madeleine Grealy and Philip Rowe conceived the study and secured funding. Milena Slachetka contributed to data collection and study management. Dwight Tse contributed to the study design and data analysis. All authors contributed to manuscript preparation, critically reviewed the manuscript, and approved the final version.

## Funding

The study was funded by the Sir Jules Thorn Charitable Trust and Chest Heart and Stroke Scotland.

## Ethics Statement

Ethical approval for this study was granted by the University of Strathclyde Ethics Committee (Reference: UEC20/08).

## Conflicts of Interest

The authors declare no conflicts of interest.

## Supporting information


**Figure S1:** CONSORT flow diagram.

**Table S1:** Eligibility criteria.

**Table S2:** Technology incorporated within the TERG rehabilitation programme.

## Data Availability

The data supporting the findings of this study will be made openly available through the University of Strathclyde PURE repository upon publication of the article. The following DOI will be activated upon acceptance of publication: https://doi.org/10.15129/f62d8a1a‐b2a4‐4a19‐b732‐a1b402d40835.
