# Supplementary material for: Intensive, Multi‐Technology Rehabilitation Programme for Chronic Stroke Survivors: Outcomes of a Phase I Single‐Arm Repeated‐Measures Study
Source: Physiother Res Int. 2026 Aug 19;31(4):e70321. doi: 10.1002/pri.70321 (PMC13488509; doi:10.1002/pri.70321)
Supplement: Supplementary file 1 [file PRI-31-e70321-s001.docx]

Table S2: Technology incorporated within the TERG rehabilitation programme

| Rehabilitation target | Examples of technologies used |
| --- | --- |
| Gait and balance training | Instrumented treadmill with virtual reality and falls arrest, balance trainer and extended reality balance activities. |
| Upper-limb motor training | Hand rehabilitation device, cable resistance system, robotic upper-limb devices and mirror therapy. |
| Lower-limb strengthening | Functional squat trainer and power-assisted exercise equipment. |
| Aerobic and whole-body exercise | Power-assisted rower, cross-cycle trainer, seated climber and static cycle. |
| Cognitive and communication activities | Large touchscreen tablet with rehabilitation applications. |

A detailed list of commercial devices used within each rehabilitation domain is available from the corresponding author on reasonable request.
