# Supplementary material for: Intensive, Multi‐Technology Rehabilitation Programme for Chronic Stroke Survivors: Outcomes of a Phase I Single‐Arm Repeated‐Measures Study
Source: Physiother Res Int. 2026 Aug 19;31(4):e70321. doi: 10.1002/pri.70321 (PMC13488509; doi:10.1002/pri.70321)
Supplement: Supplementary file 2 [file PRI-31-e70321-s002.docx]

Figure S1: CONSORT flow diagram

*Participants expressing interest, N=89*

Did not meet criteria, N=22

- Unable to attend regularly, n=3
- Still receiving rehabilitation, n=3
- < 12 months since stroke, n=5
- Currently unwell, n=4
- Deterioration in health, n=6
- Unable to consent, n=1

Met criteria, N=67

Consented, N=67

Withdrew prior to intervention, N=5

Intervention initiation, N=62

Withdrew before final session, N=3

- Transport, n=2
- Illness, n=1

Completed intervention and outcome measures, included in final analysis, N=59
