# Supplementary material for: Intensive, Multi‐Technology Rehabilitation Programme for Chronic Stroke Survivors: Outcomes of a Phase I Single‐Arm Repeated‐Measures Study
Source: Physiother Res Int. 2026 Aug 19;31(4):e70321. doi: 10.1002/pri.70321 (PMC13488509; doi:10.1002/pri.70321)
Supplement: Supplementary file 3 [file PRI-31-e70321-s003.docx]

Table S1: Eligibility criteria

| Inclusion | Exclusion |
| --- | --- |
| Diagnosis of stroke at least 12 months previously leading to mobility impairment | Absolute contradictions to physical activity |
| Currently well | Recent (6 weeks) deterioration in health |
| Not currently receiving rehabilitation | Recent (6 weeks) change in medication |
| Able to attend the programme at least twice a week | Currently unwell |
| Able to follow instructions in English and follow provide verbal and/or written feedback |  |
